# Inflammation‐Triggered Supramolecular Nanoplatform for Local Dynamic Dependent Imaging‐Guided Therapy of Rheumatoid Arthritis

**DOI:** 10.1002/advs.202105188

**Published:** 2022-01-12

**Authors:** Luoyuan Li, Xuelong Wang, Rongyao Gao, Bei Zhang, Yuxin Liu, Jing Zhou, Limin Fu, Jian Wang

**Affiliations:** ^1^ School of Pharmaceutical Sciences Key Laboratory of Bioorganic Phosphorous Chemistry & Chemical Biology (Ministry of Education) Tsinghua University Beijing 100084 P. R. China; ^2^ The Eighth Affiliated Hospital Sun Yat‐sen University Shenzhen Guangdong 518033 P. R. China; ^3^ Department of Chemistry Renmin University of China Beijing 100872 P. R. China; ^4^ Department of Chemistry Capital Normal University Beijing 100048 P. R. China

**Keywords:** inflammation‐triggered disassembly, local dynamic dependent imaging, multi‐targeted therapy, rheumatoid arthritis, supramolecular nanosystem

## Abstract

The aging of population has resulted in a significant increase in the prevalence of rheumatoid arthritis (RA), which is a persistent and recurrent synovial inflammation caused by abnormal immune activation. Herein, the authors designed an inflammation‐triggered disassembly (ITD) nanoplatform by a supramolecular assembly method, which controls the decomposition and drug release through changes in cytokine concentrations and redox potentials during the onset of arthritis, and its dual‐targeted synergistic effect on collagen‐induced arthritis (CIA) rats resulted in higher cell death rate and immunosuppressive rate. Meanwhile, they propose the local dynamic dependent imaging (LDDI) technology to diagnose the disease status, which may produce corresponding changes with the fluctuation of inflammatory activity and improve the accuracy of dual‐target therapy by monitoring the synovial changes through in situ photoactivation of the second near infrared light (NIR‐II). Very importantly, histological analysis shows that ITD strategy relieved joint destruction and cartilage degeneration and its clinical score is similar to that of the healthy group. Their work provides an effective strategy for the early diagnosis and treatment of acute and chronic inflammation diseases, which can interfere to abnormal immune activation, rather than affecting the normal function of immune system.

## Graphical abstract

The supramolecular nanosystem (ITD nanoplatforms) with tunable retention time and in situ photoactivating performance is designed for local dynamic dependent imaging‐guided integrated therapy of rheumatoid arthritis. The strategy can not only target multiple signaling pathways to surmount multidrug resistance, but also enhances the diagnosis accuracy for the severity of RA symptoms by monitoring the fluctuation of inflammatory activity.

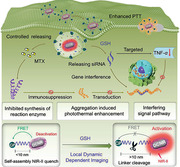

## 1 Introduction

Rheumatoid arthritis (RA) is a widespread and fatal autoimmune disease, which can lead to joint injury and even disability.^[^ ^1^, ^2^, ^3^, ^4^, ^5^ ^]^ The pathogenesis of RA involves a variety of cytokines and molecules, and its exact etiology is still unclear.^[^ ^6^, ^7^, ^8^, ^9^, ^10^, ^11^ ^]^ The treatment of RA mainly depends on non‐steroidal anti‐inflammatory drugs (NSAIDs) and disease‐modifying anti‐rheumatic drugs (DMARDs) and glucocorticoids,^[^ ^12^, ^13^, ^14^, ^15^, ^16^, ^17^, ^18^ ^]^ which mainly focus on disease remission (alleviating pain and swelling), inhibiting inflammatory response and maintaining daily physical function.^[^ ^19^, ^20^, ^21^ ^]^ But the efficacy of existing drugs is largely limited by their low bioavailability, low specificity, and high resolution rate, these drugs usually require frequent and high doses, but have high‐risk side effects. In recent years, significant achievements have been made in the development of biological agents and highly efficient inhibitors of tumor necrosis factor alpha (TNF*‐α*),^[^ ^22^, ^23^ ^]^ interleukin‐1 (IL‐1), and IL‐6.^[^ ^14^, ^24^, ^25^, ^26^ ^]^ Kishimoto, Ohshima, Rose‐John, and others revealed that antibodies play a critical role in RA treatment by blocking signaling pathway through binding with IL‐6 or its receptor, but this strategy also weakens the regulation of basic immune system activities.^[^ ^27^, ^28^, ^29^ ^]^ On the basis of the clinical trial data of RA patients, Feldman and Maini found that anti TNF‐IgG1 monoclonal antibody could interfere with the binding of TNF‐*α* to its receptor, thus weakening the function of the immune system.^[^ ^30^, ^31^, ^32^ ^]^ However, clinical data also show that many patients still have no physical response after taking TNF*‐α* or IL‐blockers, and this treatment is accompanied by high costs and risk of infection. In addition, as first‐line drugs for RA, methotrexate (MTX, a kind of disease‐modifying anti‐rheumatic drugs [DMARDs]) can improve symptoms and slow down the disease progression. However, many patients still have poor response to MTX and other clinical factors for a long time,^[^ ^33^, ^34^ ^]^ which can also induce serious side effects, such as severe liver injury and poor efficacy.^[^ ^35^, ^36^, ^37^, ^38^, ^39^ ^]^

At the same time, not only is the cost of preparing corresponding biological agents too high, but also the cytotoxicity of cytokine inhibition is still highly unpredictable. Although intra‐articular administration can improve bioavailability and reduce systemic toxicity, intra‐articular drug injection often leads to rapid clearance, which seriously affects the drug efficacy. In conclusion, more in‐depth study of the complex inflammatory microenvironment may help to find new effective methods for the treatment of RA.^[^ ^40^, ^41^, ^42^, ^43^ ^]^ The use of small interfering RNA (siRNA) to selectively reduce the production of pro‐inflammatory tumor necrosis factor has aroused considerable interest in the treatment of RA. Various methods and compositions have been explored to optimize siRNA nanocarriers to solve the problems of short half‐life, poor permeability of target tissue, poor cellular uptake, and potential immunogenicity. Although nanocarriers with high siRNA encapsulation efficiency show effective therapeutic potential in animal models, many nanocarriers still have high burst release in short circulations. Therefore, a multifunctional formulation is highly demanded, which can not only maintain high encapsulation and therapeutic efficiency against RA,^[^ ^44^, ^45^, ^46^ ^]^ but also avoid the siRNA burst release as much as possible.

In the other hand, traditional optical probes for in vivo imaging usually have limited penetration depth and spatial resolution.^[^ ^47^ ^]^ The non‐specific imaging mode make these probes produce continuous photoluminescence in systemic internal circulation, which result in poor imaging accuracy at the pathological site. Furthermore, traditional optical imaging methods with higher spectral crosstalk and self‐quenching are difficult to distinguish by the severity of symptoms during the disease diagnosis. Considering the interference caused by the complicated pathological microenvironments of RA, the biomedical application of optical probes in RA diagnosis needs to be further investigated for improving the accuracy of in vivo imaging and the distinguishability of the disease status.

**Figure 1:**
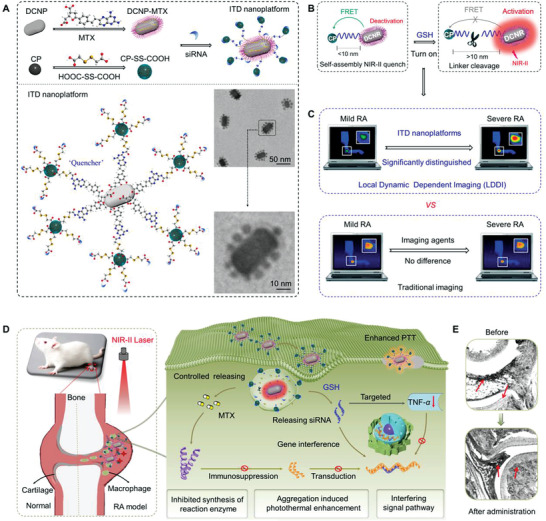
Treatment strategies and local dynamic dependent imaging method for RA. A) The structure and TEM photos of ITD nanoplatforms. B) Illustration of light up process of ITD nanoplatform by decreasing Förster resonance energy transfer (FRET) effect between CPs (serves as a “quencher” in NIR‐II imaging) and DCNR. C) Comparison of LDDI and traditional imaging mode on RA model. D) Schematic illustration of multi‐targeted treatment strategy for RA via inhibiting synthesis of reaction enzyme, breaking macrophage membrane by enhanced PTT and interfering the TNF‐*α* pathway. E) Morphological changes of rat joints after administration by multi‐targeted strategy, and red arrows indicate the morphological change of synovium.

Herein, we report a new inflammation‐triggered disassembly (ITD) of nanoplatform accompanied by local dynamic dependent imaging (LDDI) to treat RA efficiently and cooperatively (**Figure** **1**A,D). In this design, the in situ disassembly process is skillfully accomplished by the cleavable bisulfide bond (S—S) between the downconversion nanorod (DCNR) and conjugated polymer nanoparticles (CPs). In the local dynamic response to specific inflammatory milieus (Figure S1, Supporting Information), endogenous glutathione (GSH) breaks the S—S bond to ensure the light‐up activation of ITD nanoplatform (Figure 1B). It is worth noting that the dual‐targeted strategy not only interferes with the binding of TNF*‐α* to its receptor, but also directly breaks the macrophage membrane. To monitor the therapeutic effect, we further propose a special LDDI method (Figure 1C), which is expected to greatly enhance the optical contrast and reliability while suppressing the background signal in vivo. Meanwhile, it can maintain the normal function of immune system and the morphological changes of rat joints after treatment are shown in Figure 1E. Our experimental results imply that the local‐dynamic disassembly process may be closely related to the severity of RA, which offers a theoretic basis for the development of the next generation of RA diagnostic/therapeutic agents. Intra‐articular local dynamic drug release, that is, the matching between drug release and disease activity, is worthy of further exploration.

## 2 Results

### 2.1 Synthesis and Characterization of ITD Nanoplatform

**Figure 2:**
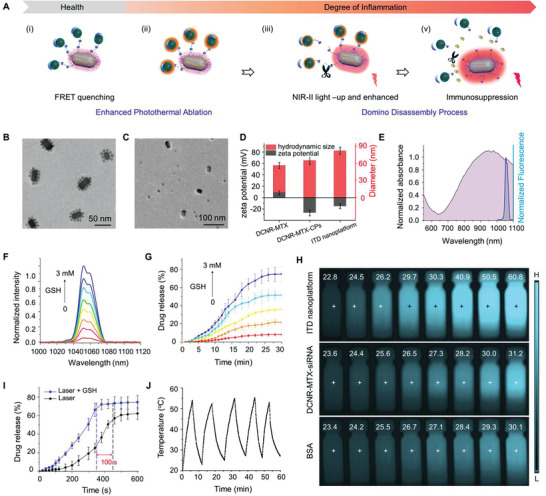
ITD nanoplatform and its physicochemical and optical properties. A) The Domino disassembly process of the ITD nanoplatforms. The TEM image of ITD nanoplatforms in phosphate buffer (PBS) (B) and in GSH (3 mm) solution (C), size distribution and zeta potentials (D), and UV–Vis–NIR spectra of ITD nanoplatforms (E). Fluorescence spectra (F) and drug release profile (G) of ITD nanoplatforms in different GSH concentrations. H) Photothermal imaging of ITD nanoplatforms, DCNR‐MTX/siRNA aqueous suspensions (150 µg mL^−1^), and BSA under 1064 nm‐light irradiation. I) Drug release profile of ITD nanoplatforms in PBS (black line) and 3 mm GSH (blue line) solution under laser irradiation. J) Photothermal stability of ITD nanoplatforms by irradiation for 60 min (five cycles of laser on/off).

ITD nanoplatforms were prepared by the supramolecular assembly method, which include multi‐step chemical synthesis and surface modification for further domino disassembly process (**Figure** **2**A). Core–shell–shell down‐conversion nanorods (NaGdF_4_:7%Nd@NaYF_4_: 1%Nd @NaYF_4_) were synthesized through a layer‐by‐layer procedure (Figure S2, Supporting Information).^[^ ^48^ ^]^ The size and morphology of the nanoparticles were measured by transmission electron microscopy (TEM, Figure S3, Supporting Information), and the columnar shape was shown with an average length of 39 nm and a width of 28 nm. The crystal structure was determined to be hexagonal phase by X‐ray powder diffraction (XRD, Figure S4, Supporting Information). Under 808 nm laser irradiation, the fluorescence characteristics of core–shell–shell DCNR were measured by fluorescence spectrometry. The emission characteristics of Nd^3+^ showed a sharp emission band in the range of 1000–1100 nm (Figure S5, Supporting Information), which was 11 times stronger than that of core DCNR (NaGdF_4_:7%Nd, Figure S6, Supporting Information). The DCNR surface was further modified with methotrexate (MTX) to acquire immunosuppressive effect by ligand exchange. The average loading content of MTX is determined to be 11.2% by calculation with Equation (1) and the loading efficiency is determined to be 62.8% by calculation with Equation (2). The hydrodynamic diameter was also determined to be ≈48 nm. Furthermore, the CPs (≈8 nm) with obvious absorption in the second near infrared region were synthesized, and then combined with DCNRs via bisulfide bridges to create ITD nanoplatforms. siRNA was used to modify the surface of the nanoplatform to target TNF‐*α* and modulate cellular immune response. The obtained ITD nanoplatforms (DCNR‐MTX‐CPs/siRNA) are a cluster structure with a diameter of 69 nm (Figure 2B).

After the addition of reductive GSH to break the disulfide bond, nanoplatforms disassembled into dispersed nanoparticles (Figure 2C). The zeta potential was changed from +8.3 mV of DCNR into −26.8 mV of DCNR/MTX, then to −15.2 mV of ITD nanoplatform (Figure 2D), and the corresponding hydration diameter was also determined by dynamic light scattering (DLS) measurement. The UV–vis–NIR absorption spectrum of the nanoplatforms showed a strong absorbance in the range of 800–1100 nm, which came from CPs (Figure 2E). Moreover, the characteristic NIR‐II fluorescence spectra of ITD nanoplatform were also measured, but its intensity was almost negligible. The quenching of the NIR‐II fluorescence could be attributed to Förster resonance energy transfer (FRET) and distance dependence between DCNR and CPs. Therefore, the NIR‐II fluorescence was further measured by adding GSH, and the intensities enhanced significantly with the increase of GSH concentration (Figure 2F). This phenomenon can be attributed to the fracture of DCNR and CPs connectors, which led to the disintegration of ITD nanoplatforms and eventually reduced the effect of FRET. Further measurement of the payload release curve by increasing the GSH concentration (Figure 2G) showed that the release content at 3 mm GSH was even as high as ≈76%, which might be caused by the decomposition of nanoparticles under the action of target molecules.

The photothermal conversion performance of ITD nanoplatforms was recorded by infrared thermal imager under 1064 nm laser irradiation of 300 and 200 mW cm^−2^, respectively. DCNR‐MTX/siRNA and bovine serum albumin (BSA) were used as controls. Thermal imaging showed that the color of the ITD nanoplatform aqueous solution (150 µg mL^−1^) changed from black (corresponding to low temperature) to bright blue (corresponding to high temperature) (Figure 2H). The recorded temperature changes indicated that the ITD nanoplatforms have a significantly higher photothermal effect than the DCNR‐MTX/siRNA nanoplatforms (Figure S7, Supporting Information). According to the temperature variation curves, the photothermal conversion efficiency (*η*) was evaluated to be 55.8% (Figure S8, Supporting Information). The payload release profile of siRNA was also measured, which exhibited that the release curve under laser irradiation for 800 s was significantly higher than that of DCNR‐MTX/siRNA nanoplatform and bovine serum albumin (BSA) (Figure S9, Supporting Information). Under the irradiation of 1064 nm laser at 200 mW cm^−2^, the releasing profile in 1 mm GSH solution was further measured. It showed that the saturation time was shortened by ≈100 s and the release content was significantly increased compared with the aqueous solution without containing GSH (Figure 2I). Photostability was further measured and its results showed that the photothermal conversion of the materials did not change after the 60 min of irradiation (Figure 2J). These results confirm that ITD nanoplatforms have high photothermal conversion efficiency and good photostability, and can be used as a potential phototherapeutic agent.

### 2.2 In Vitro Dynamic NIR‐II Imaging Detection and Activity Inhibition

**Figure 3:**
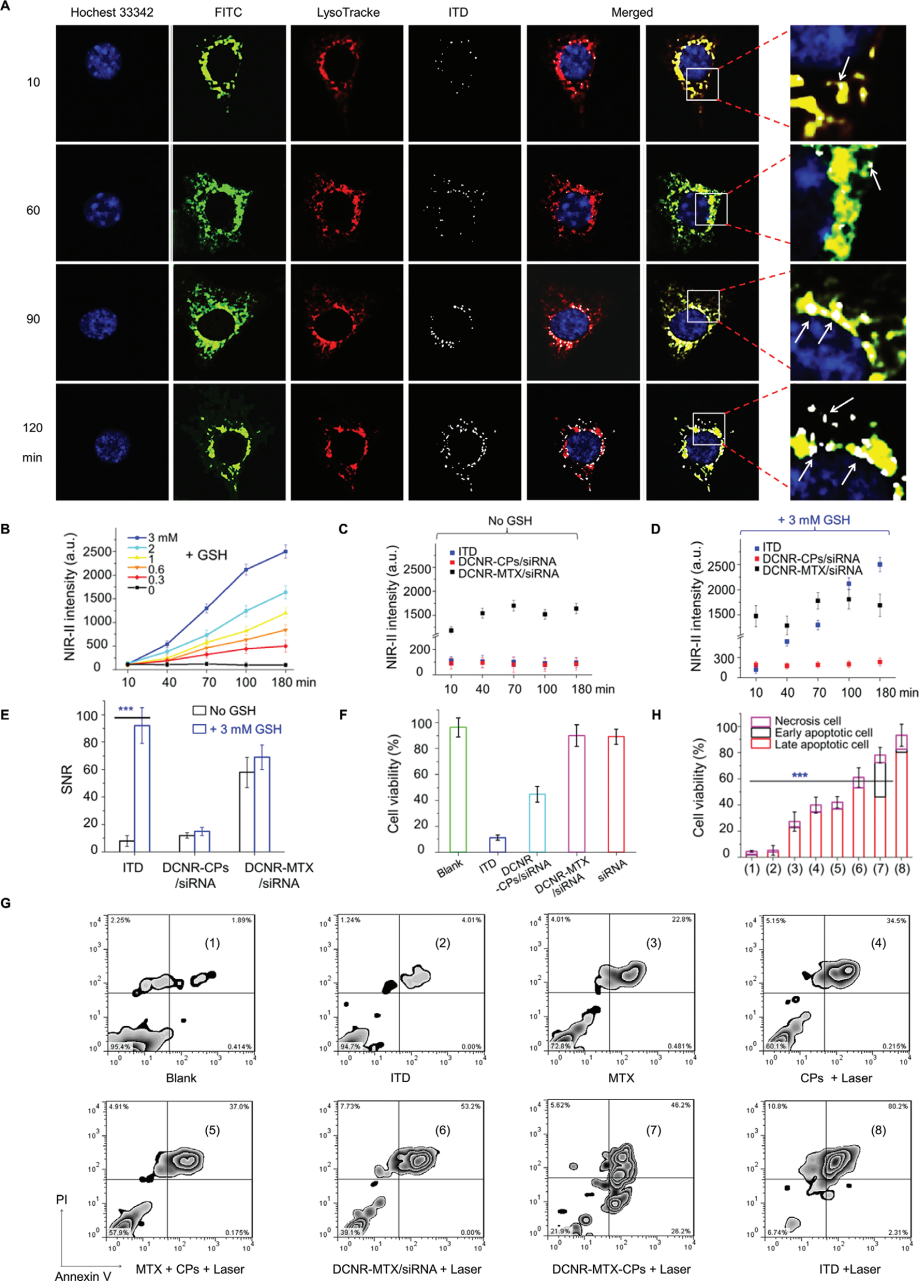
In vitro dynamic NIR‐II imaging and evaluation of inhibition effect. A) Fluorescence microscopy imaging show intracellular localization of ITD nanoplatforms in RAW 264.7 cells after incubation with addition of GSH. B) The NIR‐II intensity of cells cultured with ITD nanoplatforms in different GSH concentrations. The NIR‐II intensity of cells treated with ITD, DCNR‐CPs/siRNA, and DCNR‐MTX/siRNA (C) and in presence of 3 mm GSH (D). E) The corresponding SNR of these cells. F) The viability of cells with different treatments. Flow‐cytometric analysis of cells (G) and corresponding mortality (H) under different treatment conditions (**p < 0.01, ***p < 0.001).

The cellular uptake of ITD nanoplatforms in the RAW 264.7 cells was observed using confocal microscopy. The nucleus and lysosome were labeled with Hoechst 33 342 (blue light) and LysoTracker (red light), respectively, to monitor localization of nanoplatforms in RAW 264.7 cells. As shown in **Figure** **3**A, ITD nanoplatforms were successfully internalized by lysosome, demonstrated by the overlying of the white and red light in the presence of GSH. When the ITD nanoplatforms were added and their intracellular location monitored for 2 h, the intensity increased but dispersed in the lysosome and cytoplasm. In contrast, the ITD nanoplatform showed preferential localization in the lysosome. Quantitative co‐localization ratio of ITD nanoplatform in lysosome at different times is much higher. This specific co‐localization of ITD nanoplatforms in the endolysosomal compartment may help explain the therapeutic effect in the in vivo study.

RAW 264.7 cells were incubated to evaluate the in vitro dynamic NIR‐II imaging properties of ITD nanoplatforms (DCNR‐MTX‐CPs/siRNA), and 1060 ± 30 nm emission was set as the output signal of the acquisition channel. NIR‐II signal in the cells was negligible, which can be attributed to the FRET effect between DCNR and CPs. In order to prove the special local‐dynamic response characteristics of ITD nanoplatforms, cells were further treated with different GSH concentrations (Figure S10, Supporting Information). Compared DCNR‐CPs/siRNA and DCNR‐MTX/siRNA‐treated cells, the NIR‐II intensity of ITD‐treated cells significantly changed after adding GSH, this result was also confirmed by in vitro fluorescence imaging (Figure S11, Supporting Information). When GSH concentration was higher (3 mm), NIR‐II signal was activated in a time‐dependent manner, while NIR‐II signal recovery was weak when GSH concentration was lower (0.3 mm). Changes of NIR‐II intensity were also recorded (Figure 3B), showing time and concentration dependence. In addition, cells treated with DCNR‐CPs/siRNA and ITD nanoplatforms were used as control group. Compared with ITD‐ and DCNR‐CPs/siRNA‐treated cells, DCNR‐MTX/siRNA‐treated cells had higher NIR‐II signal at 180 min. The cytoplasm and nucleus were stained with Dil (red light) and Hochest 33 342 (blue light), respectively, and the NIR‐II signal (white) was observed in the cytoplasm. Comparing DCNR‐CPs/siRNA and DCNR‐MTX/siRNA‐treated cells (Figure 3C,D), the NIR‐II intensity of ITD‐treated cells significantly changed. Meanwhile, the increase of signal‐to‐noise ratio (SNR) in the ITD‐treated group was more obvious than that in control groups (Figure 3E). These results demonstrate that ITD nanoplatforms with GSH specific response can achieve the non‐invasive quantitative analysis of target analytes by locally and dynamically activating NIR‐II signal.

In order to show that ITD nanoplatforms have better in vitro synergistic therapeutic effect, the viability of RAW 264.7 cells cultured under different conditions (PBS, ITD, DCNR‐CPs/siRNA, and DCNR‐MTX/siRNA) was further tested with Trypan blue (Figure S12, Supporting Information). The results showed that the viability of ITD‐treated cells (9.71%) was significantly lower than that of DCNR‐CPs/siRNA (46.33%) and DCNR‐MTX/siRNA‐treated cells (90.84%) (Figure 3F). The viability of RAW 264.7 cells treated with siRNA was evaluated under 1064 nm‐laser irradiation for 600 s, showing that the viability of only siRNA‐treated cells (89.05%) was similar with that of DCNR‐MTX/siRNA‐treated cells. We also analyzed the cells with different treatments by flow cytometry (Figure 3G), and there were significant differences in the percentage of living cells. In contrast to MTX (27.2%) and CPs (39.9%) treatment groups, the cell mortality of ITD‐treated group was as high as 93.26%, which was also significantly higher than other groups (Figure 3H). Only siRNA‐treated group was further evaluated, showing that the cell inhibitory rate was 45% (Figure S13, Supporting Information), which is significantly lower than that of the ITD‐treated group. These data indicate that the ITD nanocomposites with high drug loading and targeting specificity exhibit high inhibition in vitro.

### 2.3 In Vivo NIR‐II Imaging on CIA Model Rats

**Figure 4:**
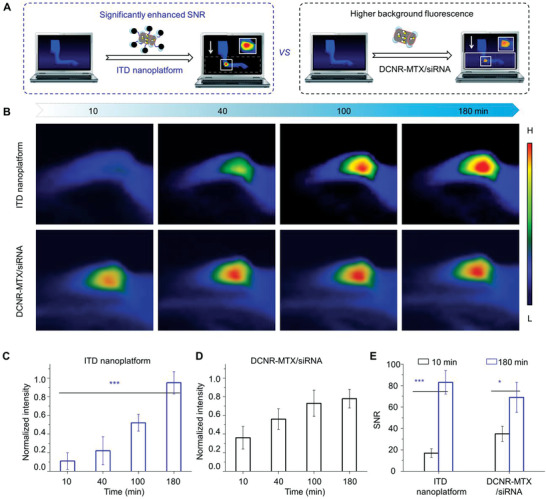
In vivo NIR‐II imaging on CIA model rats. A) Schematic illustration of comparative analysis on NIR‐II imaging. The NIR‐II imaging of rat joints treated with ITD nanoplatforms and DCNR‐MTX/siRNA (B), and corresponding intensity (C,D) of rat joints at different times post‐injection. E) The corresponding SNR of rat joints treated with these conditions (**p < 0.01, ***p <0.001).

Next, we quantitatively studied the correlation between endogenous GSH level and NIR‐II imaging of ITD nanoplatforms. The CIA model was established in Wistar rats according to the reported method.^[^ ^49^ ^]^ The CIA rats were administrated with ITD nanoplatforms via intra‐articular injection for further NIR‐II imaging, and the other group of rats treated with DCNR‐MTX/siRNA was set as control. The NIR‐II imaging of rat joints treated with ITD nanoplatforms and DCNR‐MTX/siRNA was compared (**Figure** **4**A), and the imaging effects were significantly distinct between these groups as with the light exposure time increasing (Figure 4B). The NIR‐II signal intensities of the test group did not change significantly in 40 min (Figure 4C), while that of the control group increased faster (Figure 4D). However, SNR of the test group enhanced significantly after 180 min of administration, and the variation (≈65.3%) was higher than that of the control group (Figure 4E). In addition, there was no significant difference in NIR‐II signal between the two groups after 10 h of administration, indicating a similar accumulation at the joint (Figure S14, Supporting Information). These results suggest that NIR‐II imaging is not only dependent on the aggregation of nanoplatforms, but also influenced by the disassembly activation triggered by an inflammation microenvironment.

### 2.4 In Vivo Local Dynamic Dependent Imaging (LDDI)

**Figure 5:**
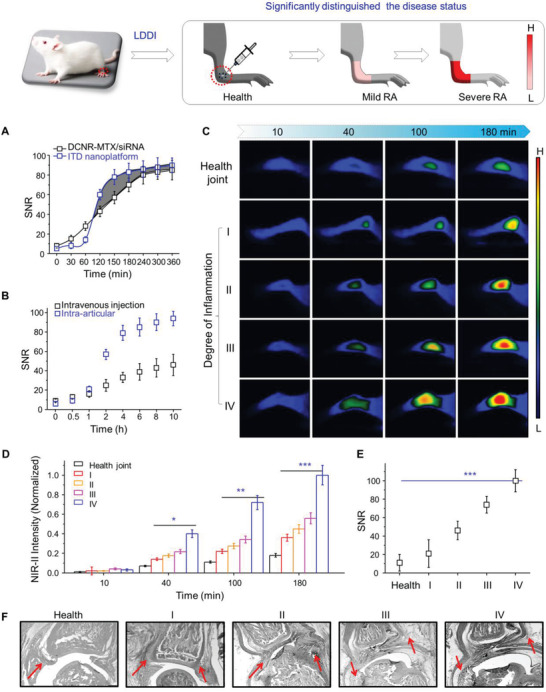
In vivo local dynamic dependent imaging. A) SNR of the rat joints treated with ITD nanoplatforms and DCNR‐CPs/siRNA. B) SNR of rat joints injected intravenously and intra‐articularly with ITD nanoplatforms. In vivo LDDI photos (C) and the NIR‐II fluorescence intensity (D) of rat joints with different degrees of inflammation. The SNR of rat joints (E) and corresponding histopathology (F) after treated with these conditions. Note: Red arrows indicating the morphological change of synovium.

To give full play to the functions of the ITD nanoplatform, we developed a dynamic optical diagnostic method “LDDI”, which could change with the fluctuation and seriousness of inflammatory activity and enhance the optical contrast of joints by switching from “off” to “on”. We first screened out the optimal interval (2–6 h) of LDDI (**Figure** **5**A), and also found LDDI has more advantages in the biological application of local administration (intra‐articular injection) than that of systemic administration (intravenous injection) (Figure 5B). The NIR‐II imaging characteristics are not only dependent on the accumulation of ITD nanoplatforms, but also on the activation of target analytes (endogenous GSH). With the aggravation of inflammation, the NIR‐II imaging intensity showed a significant enhancement (Figure 5C), which reflects that the ITD nanoplatform can be activated by GSH in the inflammatory microenvironment (Figure 5D). We then investigated the SNR of these joint sites, which also showed a gradual improvement (Figure 5E). These joints were collected for haematoxylin and eosin (H&E) staining. With the aggravation of inflammation, the synovium was significantly thickened (Figure 5F). These results confirm that LDDI method with excellent NIR‐II imaging performance can measure the severity of RA in biological system through stimulating the response of inflammatory sites on ITD nanoplatform.

### 2.5 In Vivo Dual‐Targeted Synergistic Therapy

**Figure 6:**
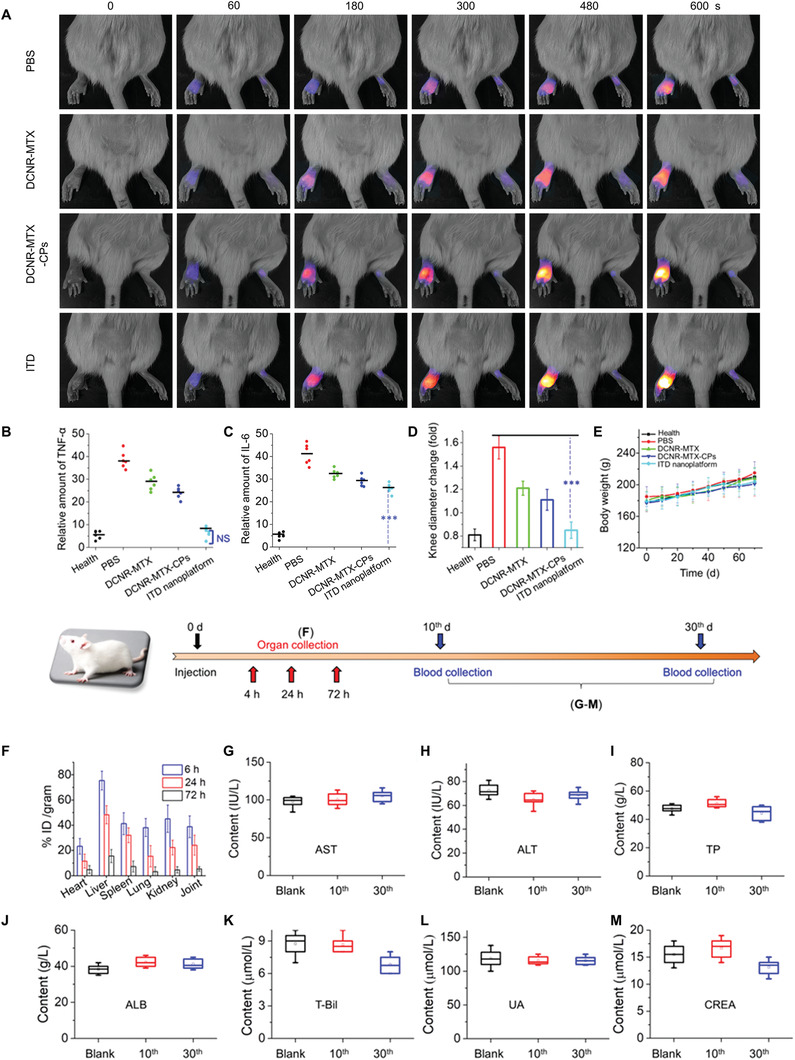
In vivo dual‐targeted synergistic therapy. A) Photothermal imaging of rat joints by treated with four different conditions. The relative amount of TNF‐*α* (B) and IL‐6 (C) with different treatments at 70 days (**p < 0.001). The changes of knee diameter (D) and body weight (E) in these groups. F) The tissue distribution of Y^3+^ uptake in the major organs of these rats. G–M) Serum biochemistry results obtained from rat injected with ITD nanoplatforms at 10th, 30th day administration and rat injected with PBS as blank group. Hepatic function markers (aspartate aminotransferase: AST, alanine aminotransferase: ALT, total protein: TP, albumin: ALB, total bilirubin: T‐Bil) and renal function markers (urea: UA, creatinine: CREA) were compared with the control group after 10 and 30 days, respectively.

To further evaluate the effect of synergistic therapy in vivo, CIA rats were divided into 4 groups (blank group [PBS]); control groups (DCNR‐MTX group; DCNR‐MTX‐CPs group); and synergistic therapy group (ITD nanoplatforms, 6.5 mg kg^−1^), with 10 rats in each group and irradiation at 200 mW cm^−2^ (**Figure** **6**A). After administration of DCNR‐MTX‐CPs and ITD nanoplatforms, the photothermal signal of the joints gradually changed from dark purple (low temperature) to bright yellow (high temperature). Obviously, the temperature changes (Δ*T*) of the ITD‐treated group showed higher than 25 °C (Figure S15, Supporting Information). The photothermal signal of rat joints only changed into orange after administration with PBS and DCNR‐MTX (Δ*T* < 10 °C). The targeting specificity of the ITD nanoplatform on CIA rats were evaluated by in vivo photothermal therapy through intravenous injection (Figure S16, Supporting Information), showing the inflammatory site with higher photothermal signal intensity. To validate the silence functionality of siRNA on TNF‐*α* cytokine, we measured the relative residues of TNF‐*α* and IL‐6 in these rats. We further investigated whether siRNA mediated ITD nanoplatforms can regulate TNF‐*α* secretion and immune response in model rats. The rats were sacrificed 1 h after treatment and the serum was processed for TNF‐*α* and IL‐6 detection by ELISA assay. Compared with PBS‐treated rats, serum TNF‐*α* secretion in ITD‐treated rats was reduced by more than 50% (Figure 6B), but serum IL‐6 secretion was reduced only ≈10% (Figure 6C), indicating that in vivo delivery of ITD nanoplatforms can inhibit TNF‐*α* secretion.

At the end of treatment, the knee diameter of rats injected with ITD nanoplatforms was significantly smaller than that of rats treated with PBS or MTX, which was equivalent to that of healthy group, indicating that the degree of joint effusion and swelling was significantly reduced. The knee diameter in the blank group has increased by 1.5 times, indicating that NIR laser irradiation had no therapeutic effect (Figure 6D). In the control groups, the knee diameters were similar to the original size, which could be attributed to the immunosuppressive effect of MTX and photothermal ablation of CPs. It is worth noting that the knee diameter in the ITD‐treated group reduced to ≈15%, which may be due to the cooperation of the immunosuppressive effect of MTX, the photothermal ablation of CPs and the selective silence effect of siRNA on TNF‐*α* cytokine. After being treated for 70 days, there was no significant difference of body weight between these groups (Figure 6E).

The tissue distribution of ITD nanoplatforms (Y^3+^ uptake %ID/gram) in the main organs of rats after administration was analyzed by inductively coupled plasma mass spectrometer (ICP‐MS). The content in liver and kidney were higher than other organs at 6 and 24 h, while the nanoplatform was almost completely metabolized after 72 h (Figure 6F). After 30 days, blood samples were collected from the experimental group (intravenous injection of ITD nanoplatforms) and the blank Wistar rats (*n* = 10) to detect their biological properties in vivo. In addition, after the injection with ITD nanoplatforms, the five hepatic function markers (Figure 6G–K) and two renal function markers (Figure 6L,M) were normal, similar to the blank group, showing that no significant hepatic and renal dysfunctions caused by the ITD nanoplatforms. After 30 days of treatment, the main organs such as heart, spleen, liver, kidney, and lung were harvested to evaluate the in vivo toxicity and H&E staining was used for histochemical study. These data showed that no significant damage was observed in these groups (Figure S17, Supporting Information), demonstrating the low toxicity of ITD nanocomposites. The pharmacokinetics of ITD nanoplatforms on CIA rats were evaluated. Free MTX was rapidly eliminated from the blood, while the ITD nanoplatform significantly prolonged circulation half‐lives (Figure S18, Supporting Information), affirming the higher bio‐effectiveness of the ITD nanoplatform. The overall results showed that ITD nanoplatforms had excellent synergistic therapeutic effect, low biological toxicity, and excellent biodegradability.

### 2.6 Preliminary Comparison of Therapeutic Effects

**Figure 7:**
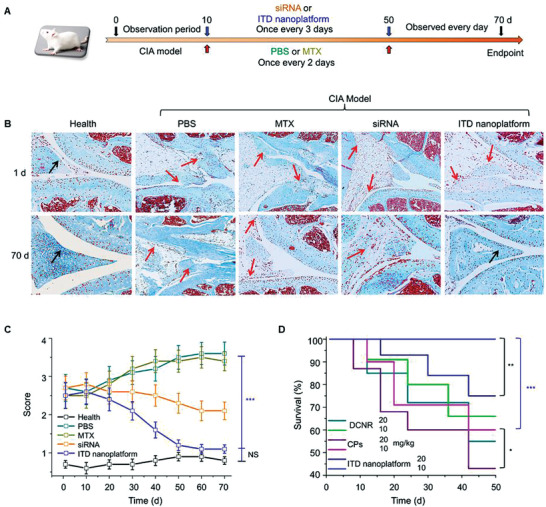
ITD nanoplatform relieves joint destruction in CIA rat model. A) Schematic illustration of dosing regimen of PBS, MTX, siRNA, and ITD nanoplatform (6.5 mg kg^−1^) for relieving joint destruction. B) Representative images of safranin‐O staining on knee sections from treated rat. Note: Red arrows indicating the morphological change of synovium. C) Clinical score of rats treated with the above treatments. D) Dose security experiment of ITD nanoplatform in Wistar rat (*p < 0.05, **p < 0.01, ***p < 0.001).

The effect of ITD nanoplatform on joint destruction was measured by using CIA model. According to the similar average clinical scores, CIA rats were divided into several groups after 30 days of immunization and 10 days of observation. ITD nanoplatforms were injected intravenously along with the negative controls of PBS, MTX, siRNA (**Figure** **7**A), and the rat joints were further sectioned for histological analysis. Sections stained with safranin‐O showed that the synovium and cartilage in ITD group recovered well, without obvious further degeneration after treatments (Figure 7B). In contrast, the cartilage in the PBS group was severely damaged and the incrassation of synovium was observed in the joints, and the situation of MTX‐treated group was similar. Less degeneration of cartilage but a little bit incrassation of synovium was observed in siRNA‐treated group. The average clinical score of PBS‐ and MTX‐treated groups increased with the development of arthritis, while the clinical score of siRNA‐treated group decreased. Significantly, the ITD‐treated group showed good efficacy on relieving joint destruction and its clinical score was similar to that of the healthy group (Figure 7C). Meanwhile, the injection dose was further optimized to ensure the biological security of ITD nanoplatform (Figure 7D). At the dose of 20 mg kg^−1^, the mortality caused by DCNRs and CP_S_ (without any surface modification) was more than 40%, while the mortality caused by ITD nanoplatform was less than 15%. At the dose of 10 mg kg^−1^, all rats treated with ITD nanoplatform survived, while the mortality of DCNRs and CPs group was still over 20%. Thus, the experimental dose (6.5 mg kg^−1^) of ITD nanoplatform lower than 10 mg kg^−1^ is safe for further application. Furthermore, the safety of ITD nanoplatform on synovial cells (HFLS) was evaluated, showing that the fatality rate of ITD nanoplatform on normal synovial cells is less than 15% (Figure S19, Supporting Information), even at a high concentration (800 µg mL^−1^).

## 3 Conclusion

In summary, we have developed a supramolecular assembly nanosystem cooperated with LDDI to enhance the immunosuppressive effect of inflammation and the cure rate of RA, which shows tunable retention time and in situ activating performance. The ITD nanoplatforms deliver drugs progressively to the location of inflammatory activity through a stepwise disassembly‐triggered manner in the synovial fluid of inflammatory joints. The high selectivity of the nanoplatform for inflammation microenvironment improved the deliver efficiency of drugs and siRNA locally, avoiding systemic exposure with long‐term toxicity. Meanwhile, our proposed LDDI method can diagnose the severity of RA symptoms by changes with the fluctuation of inflammatory activity. The in vivo optical contrast and reliability were significantly improved owing to the suppression of the background signal by reducing the FRET. Thus, our findings indicate the potential of the inflammation‐triggered disassembly strategy for effective delivery of drugs and provide a useful framework for studying transmembrane transport mechanism of siRNA delivery in the inflammation microenvironment. The biological engineering framework can also be expanded to target multiple signaling pathways to surmount the multidrug resistance and provide application prospects for imaging‐guided treatment on other acute and chronic inflammation diseases.

## 4 Experimental Section

### Materials

Gd(CH_3_CO_2_)_3_•*x*H_2_O (99.9%), Nd(CH_3_CO_2_)_3_•*x*H_2_O (99.9%), Y(CH_3_CO_2_)_3_•4H_2_O (99.99%), FeCl_3_·6H_2_O (97%), citric acid hydrate, and sodium dodecylbenzenesulfonate (SDBS, 95%) were purchased from J&K Scientific Ltd. Methotrexate (MTX, 98%) and propidium iodide (PI, 94%) were purchased from Shanghai Energy Chemical Co. Ltd. Pyrrole (99%) and dithiodiglycolic acid (96%) was purchased from Tokyo Japan Chemical Industry (TCI) Co. Ltd. NH_4_F (98%), NaOH (97%), 1‐ethyl‐3‐(3‐dimethylaminopropyl) carbodiimide hydrochloride (EDC, 97%), N‐hydroxysuccinimide sodium salt (sulfo‐NHS, 98%), and dimethyl sulfoxide (DMSO, 99.9%) were purchased from Sinopharm Chemical Reagent Co. China. 1‐Octadecene (95%), oleic acid (>95%), TNF‐*α* siRNA, 1,2‐dioleoyl‐3‐trimethylammonium‐propane (DOTAP), Hoechst 33 258 (98%), and Annexin V‐FITC apoptosis detection kit were purchased from Sigma‐Aldrich. All chemical reagents were purchased from commercial sources and used directly without further purification. Deionized water was used throughout.

### Characterization

The sizes and morphologies of ITD nanoplatforms were determined using a H‐7650B transmission electron microscope (TEM, 100 kV, HITACHI). Ultraviolet–visible–near‐infrared (UV–Vis–NIR) absorption spectra were obtained on a Shimadzu UV‐3600 spectrophotometer. Powder X‐ray diffraction (XRD) measurement was measured with a Shimadzu 7000 X‐ray diffractometer (Cu K*α* radiation, *λ* = 1.54 Å). Dynamic light scattering (DLS) experiment was conducted on a light scattering analyzer (Zetasizer Nano ZS90, Malvern). The luminescence spectra were measured by using a fluorescence spectrophotometer (FLS980, Edinburgh Instruments). These photoluminescence studies were conducted at room temperature. The fluorescence spectra were carried out on an F‐4600 spectrophotometer (HITACHI). The imaging photos were acquired with a Nikon CCD Camera.

### Synthesis of the ITD Nanoplatforms

In the typical process,^[^ ^50^ ^]^ Gd(CH_3_CO_2_)_3_ (0.372 mmol), and Nd(CH_3_CO_2_)_3_ (0.028 mmol) were added into a mixture of oleic acid (3 mL), 1‐octadecene (7 mL), and water (2 mL). Then, the mixture was heated to 160 °C for 1 h to form a homogeneous solution. Thereafter, NaOH (1 mmol) and NH_4_F (1.6 mmol) were added into the solution after naturally cooled down to room temperature under vigorous stirring. The mixture was further heated to 100 °C for 20 min and then rapidly heated to 295 °C under argon atmosphere. After reacted for 1 h, the solution was naturally cooled down to room temperature. The NaGdF_4_:Nd nanoparticles were collected and washed with cyclohexane for several times, and dispersed in cyclohexane for further use.

In the similar process, Gd(CH_3_CO_2_)_3_ (0.396 mmol) and Nd(CH_3_CO_2_)_3_ (0.004 mmol) were added into a mixture of oleic acid (3 mL) 1‐octadecene (7 mL) and water (2 mL). Then, the mixture was heated to 160 °C for 1 h to form a homogeneous solution and cooled naturally to room temperature. Thereafter, NaOH (1 mmol) and NH_4_F (1.6 mmol) were added to the solution along with the as‐prepared NaGdF_4_:Nd core nanoparticles (5 mL in cyclohexane) under vigorous stirring. Subsequently, the resulted mixture was heated to 100 °C for another 20 min and then rapidly heated to 295 °C and stirred under argon atmosphere for 2 h. The core–shell nanoparticles were collected by centrifugation and washed for several times, and then dispersed in cyclohexane.

The synthetic process of tri‐layered core–shell–shell nanomaterials is the same as that of core–shell nanomaterials. The as‐prepared NaGdF_4_:7%Nd@NaYF_4_:1%Nd core–shell nanomaterials (5 mL in cyclohexane) were utilized as seeds and Y(CH_3_CO_2_)_3_ (0.4 mmol) was used as the outermost shell precursor. The NaGdF_4_:7%Nd@NaYF_4_:1%Nd@NaYF_4_ tri‐layered nanorods (DCNRs) were dispersed in cyclohexane for further use. The surface of DCNRs was further modified through ligand exchange to replace OA with methotrexate (MTX). Cyclohexane (15 mL), ethanol (15 mL), and MTX (0.5 g) were added into a flask containing 0.1 g of DCNRs and then stirred at room temperature for 24 h. The product (DCNR‐MTX) was collected by centrifugation (5000 rmp) for 10 min and then washed with ethanol for several times. The average loading content of MTX is calculated with Equation (1) and the loading efficiency is calculated with Equation (2):

(1) $$\mathrm{MTX}\;\;\mathrm{loading}\;\;\mathrm{content}\left(\%\right)=M_{\text{MTX}\;\text{in}\;\text{nanoplatform}}/M_{\mathrm{nanoplatform}}\times 100\%$$

(2) $$\begin{array}{ccc} & & \mathrm{MTX}\;\;\mathrm{loading}\;\;\mathrm{efficiency}\left(\%\right)=[(\mathrm{mean}\;\;\mathrm{of}\;Abs_{\mathrm{initial}\;\;\mathrm{MTX}}\\ & & \;-\mathrm{mean}\;\;\mathrm{of}\;Abs_{\text{final}\;\mathrm{supernatant}})/\mathrm{mean}\;\;\mathrm{of}\;Abs_{\text{initial}\;\text{MTX}}] \end{array}$$

In the typical procedure,^[^ ^51^, ^52^, ^53^ ^]^ SDBS (4 mmol) was added into a mixture of H_2_SO_4_ (6 mL, 0.2 m), citric acid hydrate (1.45 mm), deionized water (12 mL), and methanol (10 mL) under agitation and then was adjusted to pH = 1 after vigorous stirring for 30 min. Pyrrole (100 µL) and equal molar ratio of FeCl_3_·6H_2_O was added to form a homogeneous solution under magnetic stirring. The resulting mixture was transferred to a 50 mL of autoclave, sealed and hydrothermally treated at 140 °C for 5 h. Next, the mixture was naturally cooled down to room temperature and the precipitates were collected by adding methanol. The product (CPs) was obtained by purifying after washing several times with methanol and water to remove other residual compounds. Finally, the product was dispersed in methanol/water solution (1:1). 200 µL of EDC/NHS (20 mg mL^−1^) and equivalent amounts of dithiodiglycolic acid were added into the solution containing CPs. The reaction continued at 50 °C for 8 h and then the product was collected by centrifugation (5000 rpm) for 10 min and washed several times with water to remove residual compounds.

The ITD nanoclusters were prepared by the supramolecular assembly method. Dithiodiglycolic acid‐modified CPs (200 mg) was added to the ethanol solution containing DCNR‐MTX (100 mg) and the reaction was continued at 50 °C for 12 h. The resulting product (DCNR‐MTX‐CPs) was collected by centrifugation (5000 rpm) for 10 min and washed several times with water to remove residual CPs. Then, 1 µm TNF‐*α* siRNA (5′GUCUCAGCCUCUUCUCAUUCCUGCT‐3′) was added to phosphate buffer (100 µL, pH 5.5) containing DCNR‐MTX‐CPs (50 mg) to further incubate at low temperature for 30 min. The final product (DCNR‐MTX‐CPs/siRNA) was separated through a low‐speed centrifugation (800 rpm).

### Photothermal Properties of ITD Nanoplatforms

Photothermal properties were measured by using FLIR E40 equipment described in previous literatures.^[^ ^54^, ^55^, ^56^ ^]^ The photothermal conversion photos of ITD aqueous solution (150 µg mL^−1^) were achieved by conjunctions with a 1064 nm laser. Temperature changes were also recorded at different time intervals. The ITD solution (150 µg mL^−1^) was placed in a specimen bottle irradiated by 808 nm laser (200 mW cm^−2^). The photothermal properties under different laser power (200, 300 mW cm^−2^) and photostability after 60 min irradiation were measured.

### Cell Viability Assay

RAW 264.7 cells (monocyte macrophage) were grown in high‐glucose Dulbecco's modified Eagle's medium (DMEM, Solarbio, Beijing) supplemented with 10% fetal bovine serum (FBS, Gibco, US). Cultures were maintained at 37 °C under a humidified atmosphere containing 5% CO_2_. 1 × 10^6^ cells/well was seeded in disposable culture dish (10 mm) for 24 h prior to the assay. The cells treated with different conditions after 48 h stained with Annexin V/PI kit and then analyzed by flow cytometry using a Beckman instrument. All these cells were provided by the Institute of Basic Medical Sciences, Chinese Academy of Medical Sciences.

### In Vivo Toxicity of ITD Nanoplatforms

Tissues (tumor, heart, spleen, liver, kidney, and lung) were harvested and fixed in paraformaldehyde and embedded in paraffin. Furthermore, these samples were sectioned and stained with H&E. The Y^3+^ uptake content in organs and tumor distribution were evaluated by ICP‐MS analysis. Blood samples were collected from the CIA rats after administration with ITD nanoplatforms and PBS (*n* = 10) on 10th and 30th day. Five important hepatic indicators (aspartate aminotransferase: AST, alanine aminotransferase: ALT, total protein: TP, albumin: ALB, total bilirubin: TBIL), and two indicators for renal functions (creatinine: CREA and urea: UA) were evaluated. Tissues were dissected from the test and control groups.

### Establishments of CIA Model Rats

Collagen‐induced arthritis (CIA) model was induced in ≈8–12 week‐old female Wistar rats (Beijing Vital River Laboratory Animal Technology Co., Ltd.) according to the operation protocol of a previous report. Chicken type II collagen in an emulsion with Freund's Complete Adjuvant was used for initial immunization, and chicken type II collagen in an emulsion with Freund's Incomplete Adjuvant was used for boosting. The emulsions were intradermally injected in the base of the tail. Autoimmune arthritis is induced in this model by immunization with an emulsion of complete Freund's adjuvant and type II collagen. Typically, the first signs of arthritis appear in this model ≈30–35 days after initial immunization. Rats were observed daily for signs of joint inflammation.

### In Vitro and In Vivo NIR‐II Imaging of ITD Nanoplatforms

For in vitro NIR‐II imaging, RAW 264.7 cells were incubated with the medium containing ITD nanoplatforms, DCNR‐CPs and DCNR‐MTX/siRNA (150 µg⋅mL^–1^), respectively. Fluorescence signal was collected at 1060 ± 30 nm exposed by an 808 nm laser and measured by an obtained digitally on a Nikon CCD camera. Cell nucleus and membrane were stained with Hoechst‐33342 and DiI, respectively. To further evaluate the in vivo NIR‐II imaging properties, rats were intra‐articular injected with ITD nanoplatforms (6.5 mg kg^−1^), and that with DCNR‐MTX/siRNA were set as the control group. The intensities and SNR of NIR‐II imaging were also recorded by InGaAs CCD excited at 808 nm.

### In Vivo Synergistic Therapeutic Effect

CIA rats were divided into 4 groups: blank group (PBS); control groups (DCNR‐MTX group; DCNR‐MTX‐CPs group). In the synergistic therapy group (ITD nanoplatforms, 6.5 mg kg^−1^), 10 rats in each group were irradiated with 200 mW cm^−2^. The photothermal signal and temperature changes (Δ*T*) of the joints after administration were also recorded by the FLIR E40 equipment. After administration on the 70th day, knees were dissected from these groups and the knee diameters of these rats were recorded. Body weights and the relative remain amount of TNF‐*α* and IL‐6 were evaluated by ELISA assay. The tissue distributions in the major organs of these rats after administration were measured by ICP‐MS.

### Statistical Analysis

All data are presented as means ± SD. Statistical analyses were completed by performing analysis of variance. *p* ≤ 0.05 was considered significant.

## Conflict of Interest

The authors declare no conflict of interest.

## Supporting information

Supporting Information

## Data Availability

The data that support the findings of this study are available from the corresponding author upon reasonable request.
