# Supplementary material for: Inflammation‐Triggered Supramolecular Nanoplatform for Local Dynamic Dependent Imaging‐Guided Therapy of Rheumatoid Arthritis
Source: Adv Sci (Weinh). 2022 Jan 12;9(7):2105188. doi: 10.1002/advs.202105188 (PMC8895155; doi:10.1002/advs.202105188)
Supplement: Supplementary file 1 — Supporting Information [file ADVS-9-2105188-s001.pdf]

WILEY-VCH

ADVANCED SCIENCE
Open Access

Supporting Information

for *Adv. Sci.*, DOI: 10.1002/advs.202105188

Inflammation-Triggered Supramolecular Nanoplatform for Local Dynamic Dependent Imaging-Guided Therapy of Rheumatoid Arthritis

Luoyuan Li, Xuelong Wang, Rongyao Gao, Bei Zhang, Yuxin Liu, Jing Zhou,* Limin Fu,* and Jian Wang**

Supporting Information

Inflammation-Triggered Supramolecular Nanoplatform for Local Dynamic Dependent Imaging-Guided Therapy of Rheumatoid Arthritis

Luoyuan Li, Xuelong Wang, Rongyao Gao, Bei Zhang, Yuxin Liu, Jing Zhou,* Limin Fu,* and Jian Wang**

*Corresponding author. Email: wangjian2012@tsinghua.edu.cn; lily98@mail.sysu.edu.cn; jingzhou@cnu.edu.cn; lmfu@ruc.edu.cn

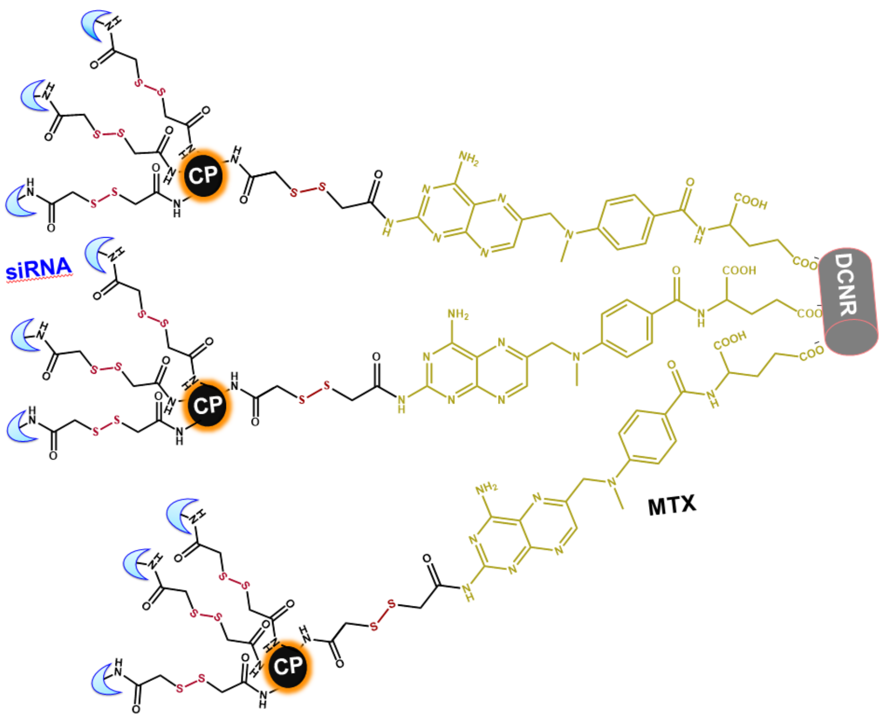

Figure S1. Chemical structure of ITD nanoplatforms.

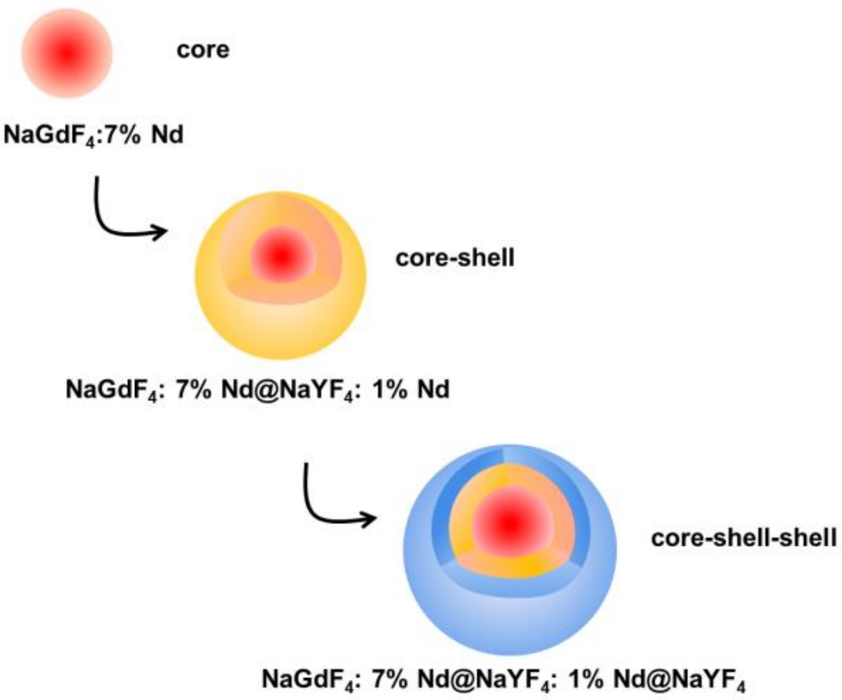

Figure S2. Procedure for the layer-by-layer epitaxial growth method of core-shell-shell nanomaterials.

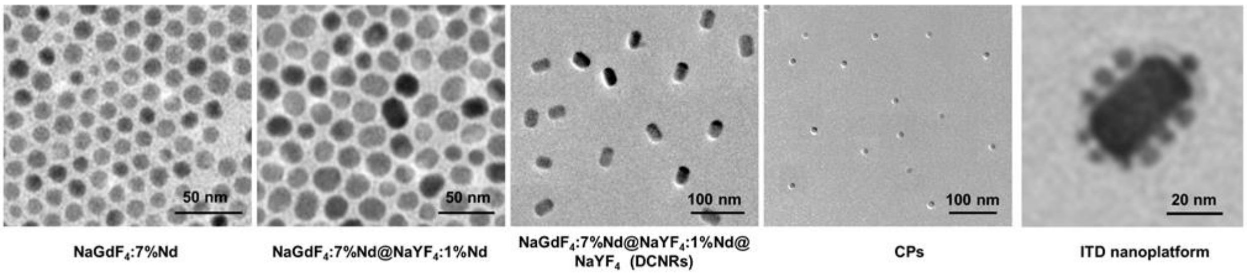

Figure S3. TEM images of $NaGdF_4$:7%Nd, $NaGdF_4$:7%Nd@$NaYF_4$:1%Nd, $NaGdF_4$:7% Nd@$NaYF_4$:1%Nd@$NaYF_4$ (DCNRs) and CPs nanocrystals.

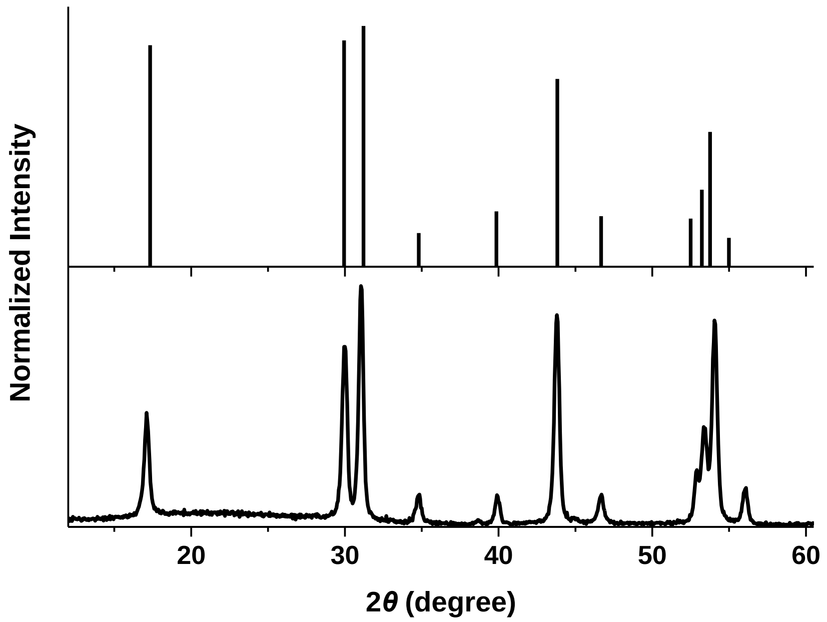

Figure S4. XRD of DCNRs and the corresponding standard card (Joint Committee on Powder Diffraction Standards file No. 16-0334) of the pure hexagonal phase

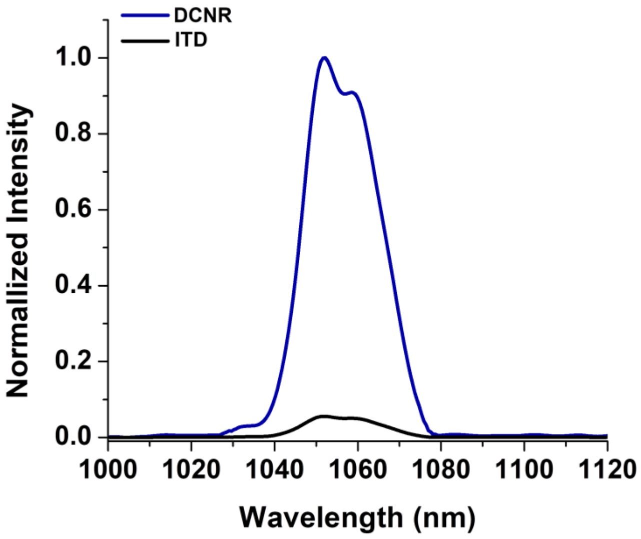

Figure S5. Fluorescence spectra of ITD (black curve) and DCNR (blue curve).

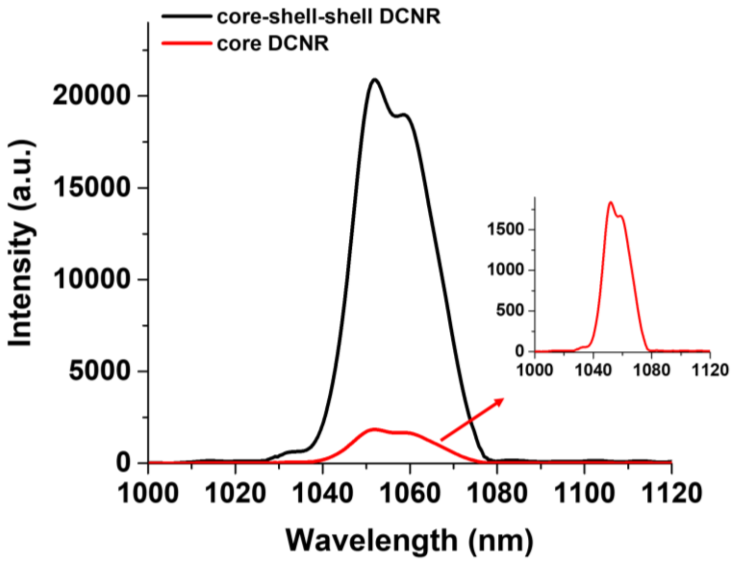

Figure R6. Fluorescence spectra of core DCNR (red curve) and core-shell-shell DCNR (black curve).

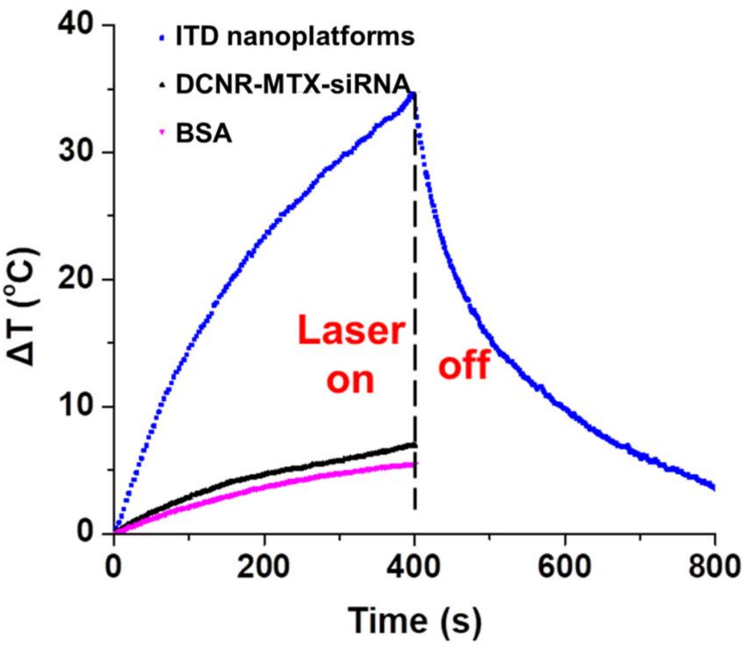

Figure S7. The photothermal conversion curves of BSA, ITD and DCNR-MTX-siRNA nanoplatforms.

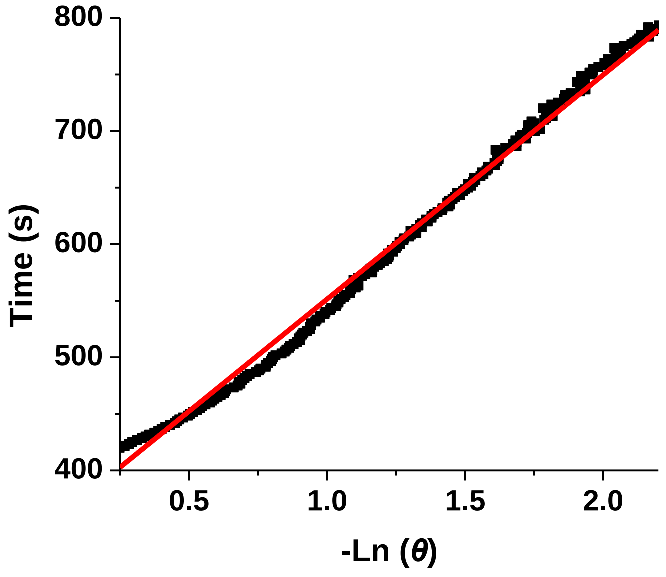

Figure S8. Plot of time against –Ln(θ) derived from the cooling period of fig. S6 when the laser was shut off. The fitting line in red follows y = 393.24 + 198.16x (R = 0.991).

$$\eta = (hA\Delta T_{\max} - H_s)/[P \times (1 - 10^{-Abs_{1064\text{nm}}})]$$

where $\Delta T_{\max}$ is the maximal temperature change, H_s is the heat associated with the light absorbance by water, P is the laser power and Abs_{1064nm} is the absorbance at 1064 nm for the

nanogels. In Eq. (1), $hA = mC_p/\tau$ is needed to introduce θ ($\Delta T/\Delta T_{max}$), a ratio of ΔT (temperature change) over ΔT_{max}, where τ is the slope of $-\mathrm{Ln}(\theta)$ *vs* time, and m and C_p are the mass and the heat capacity of water, respectively.

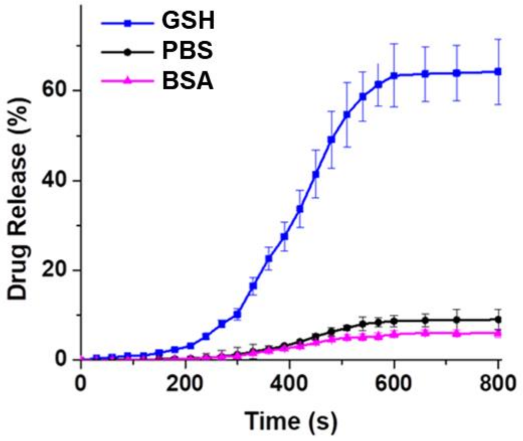

Figure S9. Drug release profile of ITD nanoplatforms in PBS, BSA and 3 mM GSH (blue line) solution under laser irradiation.

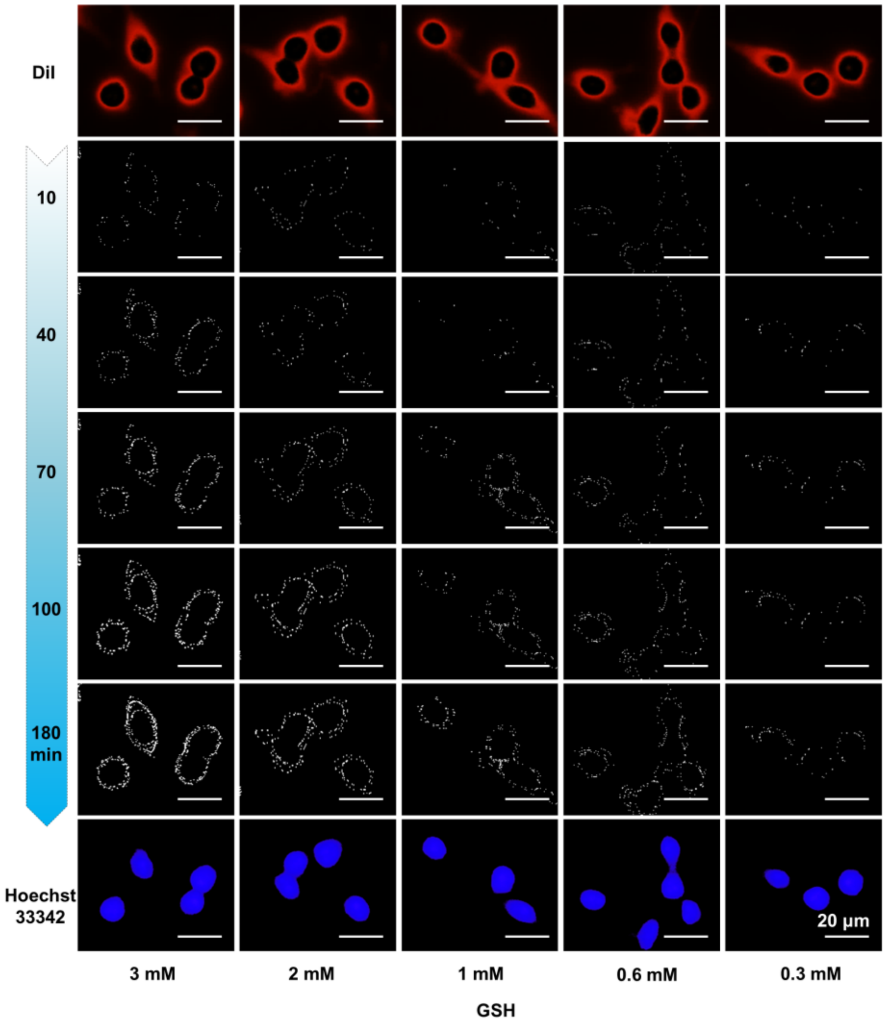

Figure S10. Fluorescence microscopic images of RAW 264.7 cells cultured with ITD nanoplatforms by adding different GSH concentrations in 180 min.

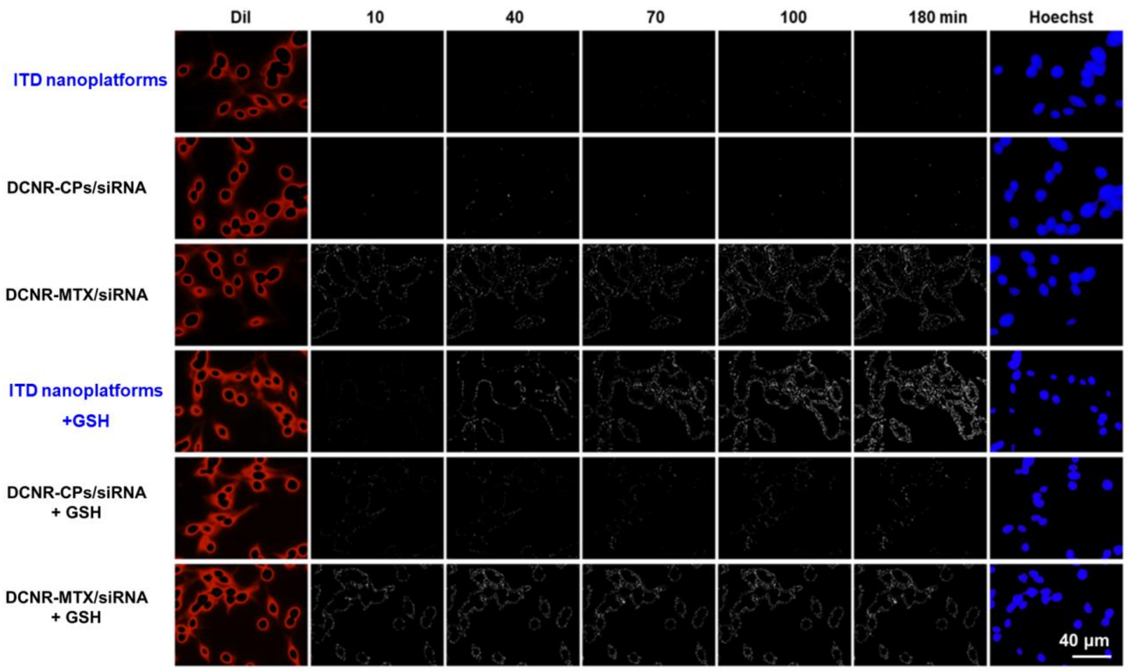

Figure S11. Fluorescence microscopic images of RAW 264.7 cells cultured with ITD, DCNR-CPs/siRNA and DCNR-MTX/siRNA and by adding an additional GSH upon 808 nm laser irradiation.

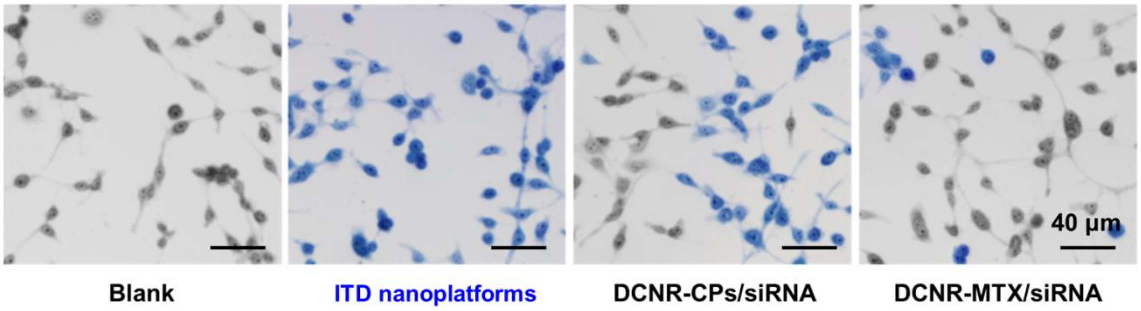

Figure S12. Bright fields photos of cells stained by Trypan blue after different treatments.

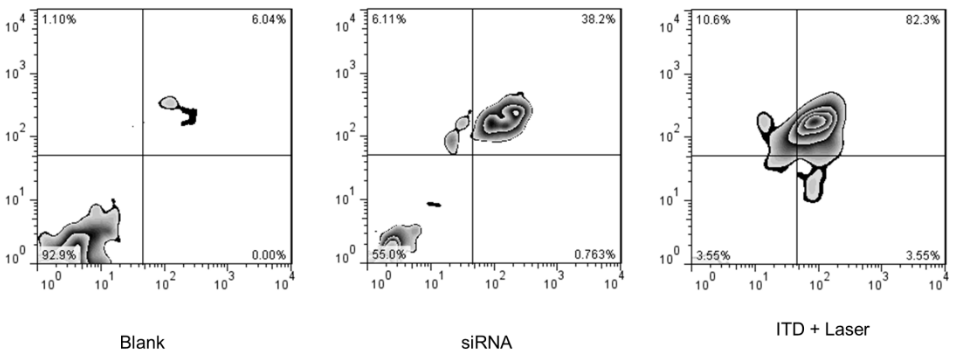

Figure S13. Flow-cytometric analysis of cells treated with different conditions.

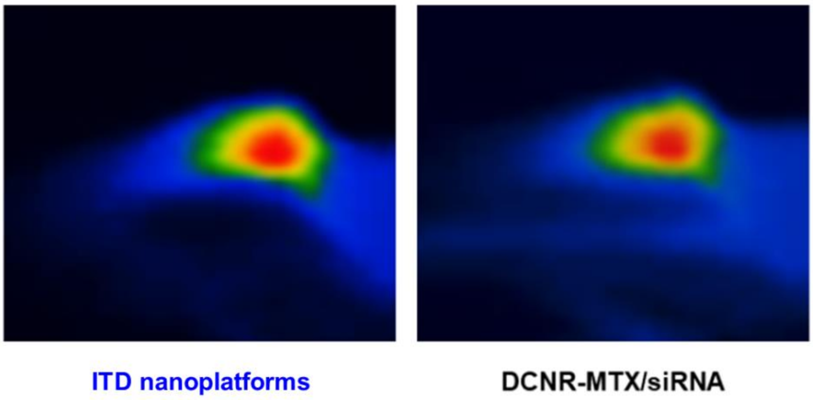

Figure S14. *In vivo* NIR-II fluorescence images of rat joints at 10 h post-injection.

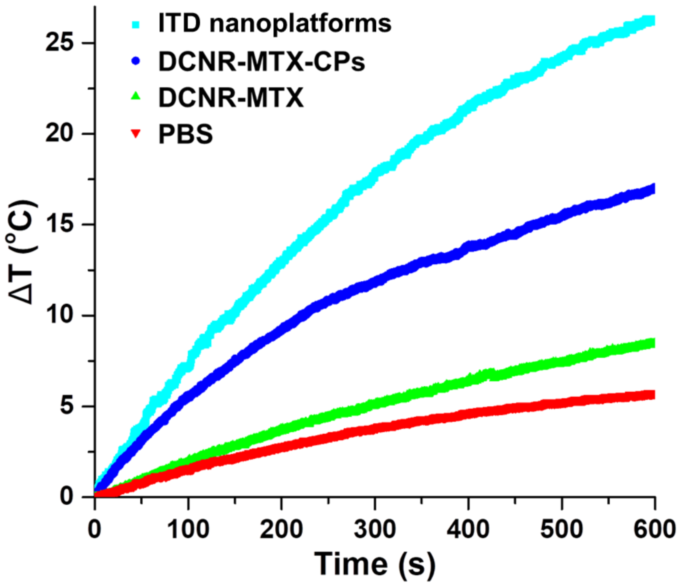

Figure S15. The temperature changes of the rat joints treated with four different conditions under NIR-light irradiation.

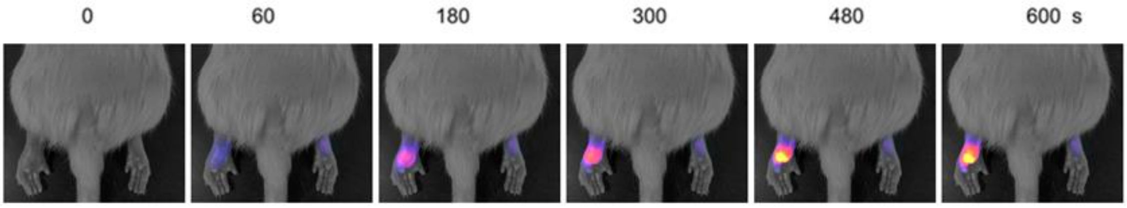

Figure R16. Photothermal imaging of rat joints by treated intravenously with ITD nanoplatforms, showing the specific selectivity of ITD nanoplatforms in CIA rats.

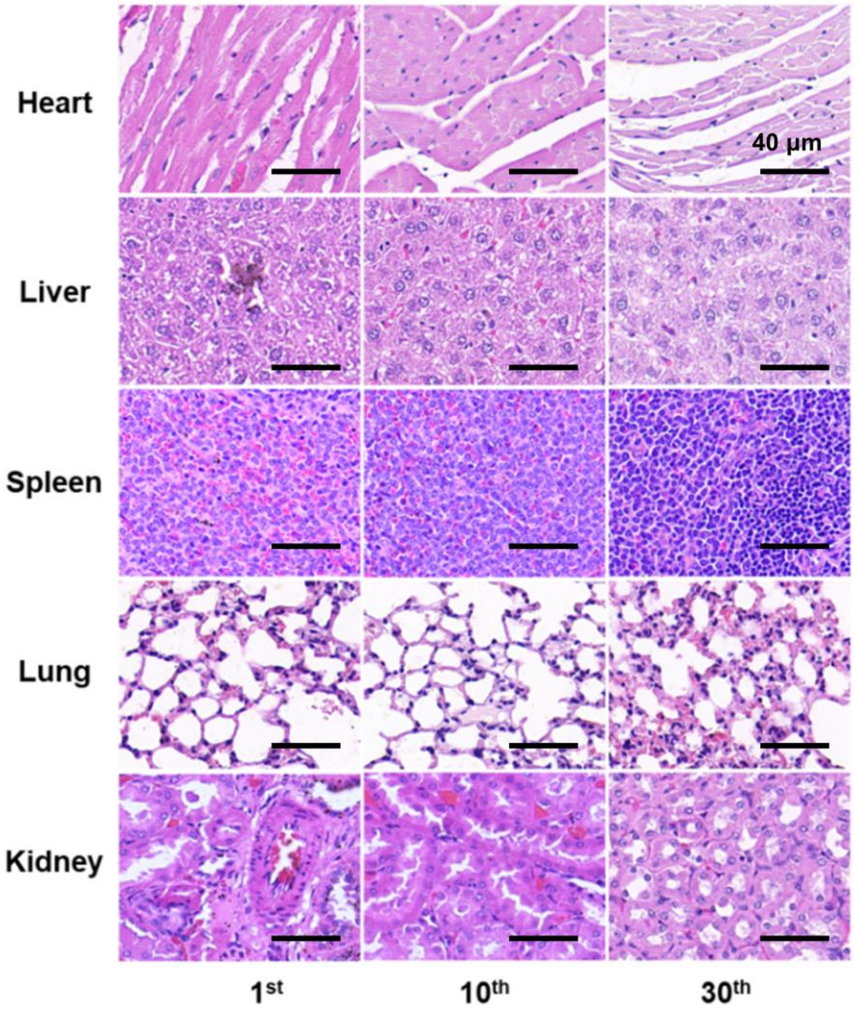

Figure S17. Microscopic images of H&E stained tissue sections of organs from the blank (1st) and the tested rats after tail vein injection with DCNR/MTX-CPs/siRNA for 30 days.

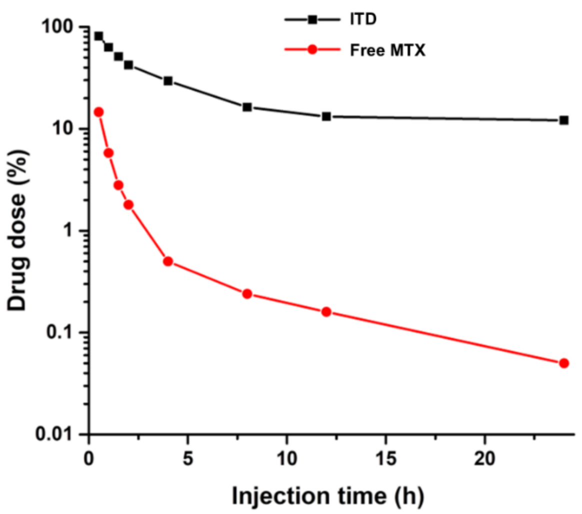

Figure S18. Blood kinetics of ITD nanoplatform and free MTX (3 mg/kg) in CIA rat (n= 4) injected intravenously.

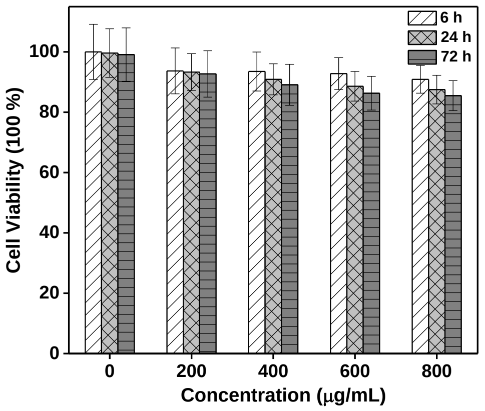

Figure S19. Synovial cell viability after incubation with ITD nanoplatform for 6 h, 24 h, and 72 h, showing that the fatality rate of ITD nanoplatform on normal synovial cells is less than 15% even at high concentration (800 μg/mL).
